# A Rare Presentation of Teratocarcinosarcoma as a Carinal Mass With Review of Literature

**DOI:** 10.1002/rcr2.70571

**Published:** 2026-04-11

**Authors:** Gittwa Kottangal, Vishnu Gopalakrishnan, Binil Salam, Avinash Murugan, Supriya Ranjith, Pramathakala Mettak, Vishnu Suresh

**Affiliations:** ^1^ Department of Pathology Aster MIMS Hospital Calicut India; ^2^ Department of Interventional Pulmonology Aster MIMS Hospital Kannur India

**Keywords:** airway obstruction, carina, endobronchial debulking, multimodality treatment, teratocarcinosarcoma

## Abstract

Teratocarcinosarcoma (TCS) is a rare, highly aggressive malignant neoplasm composed of epithelial, mesenchymal and neuroectodermal elements, occurring almost exclusively in the sinonasal region. Primary involvement of the tracheobronchial tree or carina has not been previously reported. We describe a 55‐year‐old male chronic smoker who presented with cough, progressive dyspnea and haemoptysis. Computed tomography revealed a lobulated soft‐tissue mass in the distal trachea overhanging the carina, extending into both main bronchi, causing significant airway obstruction. Bronchoscopy demonstrated greater than 80% bilateral luminal occlusion, necessitating urgent endobronchial debulking using rigid bronchoscopy, snare electrocautery, and cryotherapy. Histopathology with immunohistochemistry confirmed teratocarcinosarcoma. Positron emission tomography‐computed tomography (PET‐CT) showed metabolically active mediastinal and hilar lymph nodes. The patient was treated with chemoradiotherapy. Follow‐up bronchoscopy at 6 months showed no recurrence, and the patient remains asymptomatic under surveillance for 4 years. This case represents the first reported carinal presentation of teratocarcinosarcoma, expands the anatomical spectrum of TCS and emphasizes the value of multimodality treatment for sustained disease control.

## Graphical abstract

We report a rare presentation of teratocarcinosarcoma arising at the carina with bilateral main bronchial obstruction, resulting in critical airway compromise. Urgent bronchoscopic debulking followed by definitive chemoradiotherapy achieved sustained disease control with no recurrence at 4‐year follow‐up. This case highlights the importance of accurate histopathological diagnosis and multimodality management in unusual central airway tumours.

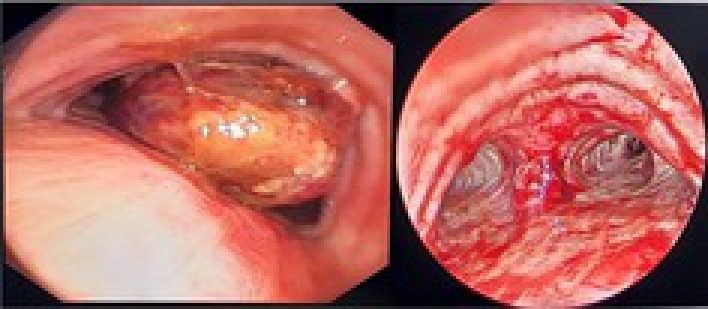

## 1 Introduction

Teratocarcinosarcoma (TCS) is an uncommon, highly aggressive malignant tumour composed of elements derived from all three germ layers—ectodermal, mesodermal and endodermal tissues. It predominantly arises from the maxillary or ethmoid sinuses and accounts for less than 1% of all sinonasal tumours [1, 2, 3]. TCS predominantly affects adult males, with a reported male‐to‐female ratio of approximately 4:1 [4].

Although rare cases have been described in the nasopharynx, oral cavity, thyroid, hypopharynx, intracranial region and female reproductive organs [5, 6, 7, 8, 9, 10, 11, 12, 13], pulmonary or tracheobronchial origin has not been previously documented. We report what appears to be the first known case of TCS originating in the carina, presenting with bilateral main bronchial involvement.

## 2 Case Report

A 55‐year‐old male, a known hypertensive and chronic smoker (smoking index 1000), presented with chronic cough, progressive dyspnea, and intermittent haemoptysis for 1 week. He consumed alcohol occasionally. General and systemic examinations were unremarkable.

**FIGURE 1:**
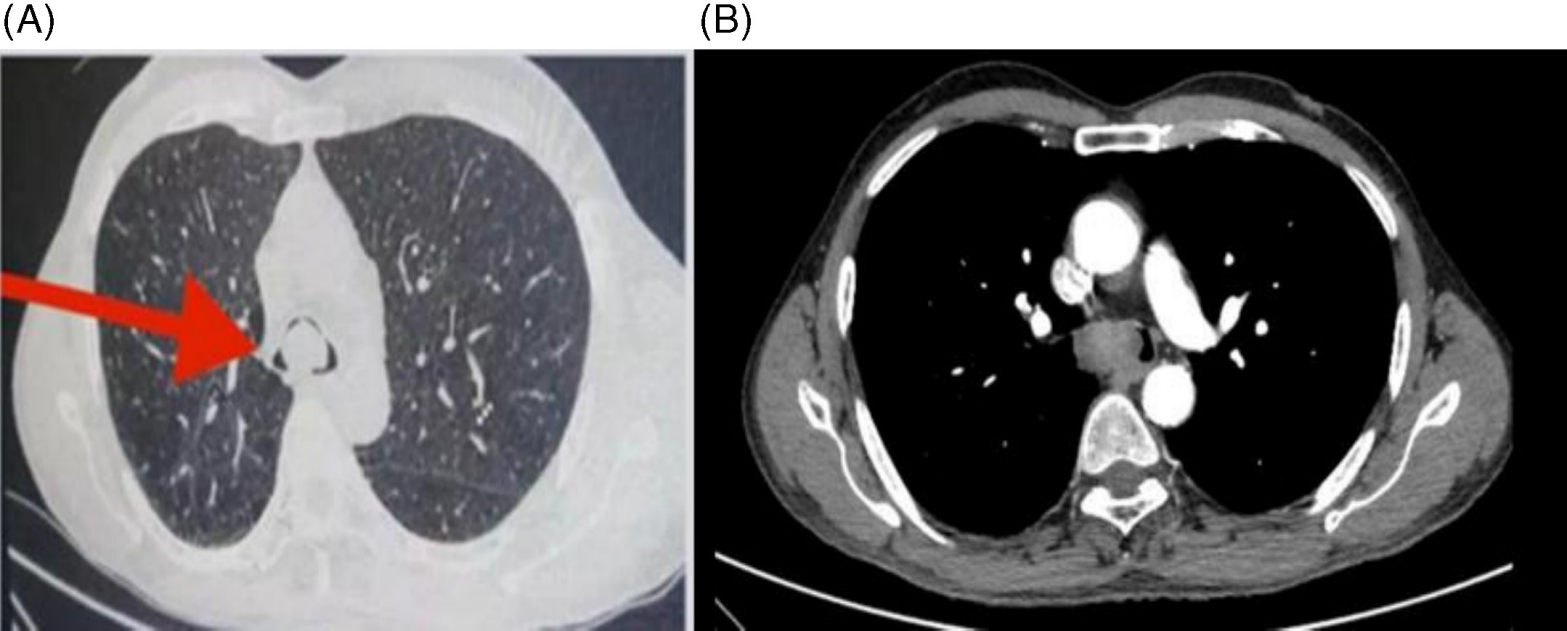
(A) The axial CT section of thorax, Lung window at the level of distal trachea. (B) The axial CT section of thorax, mediastinal window at the level of carina, the post‐contrast image. Both showed a 3 × 2.1 × 1.4 cm well marginated, lobulated, soft tissue density mass with heterogeneous post contrast enhancement at the distal trachea, extending into both proximal main bronchi.

High‐resolution CT (HRCT) of the thorax showed a well‐marginated, lobulated soft‐tissue mass measuring 3 × 2.1 × 1.4 cm at the distal trachea, projecting over the carina and extending into both proximal main bronchi, with heterogeneous post‐contrast enhancement, suggestive of a neoplastic aetiology. Figure 1A for the axial lung window view and Figure 1B for the axial mediastinal window view.

**FIGURE 2:**
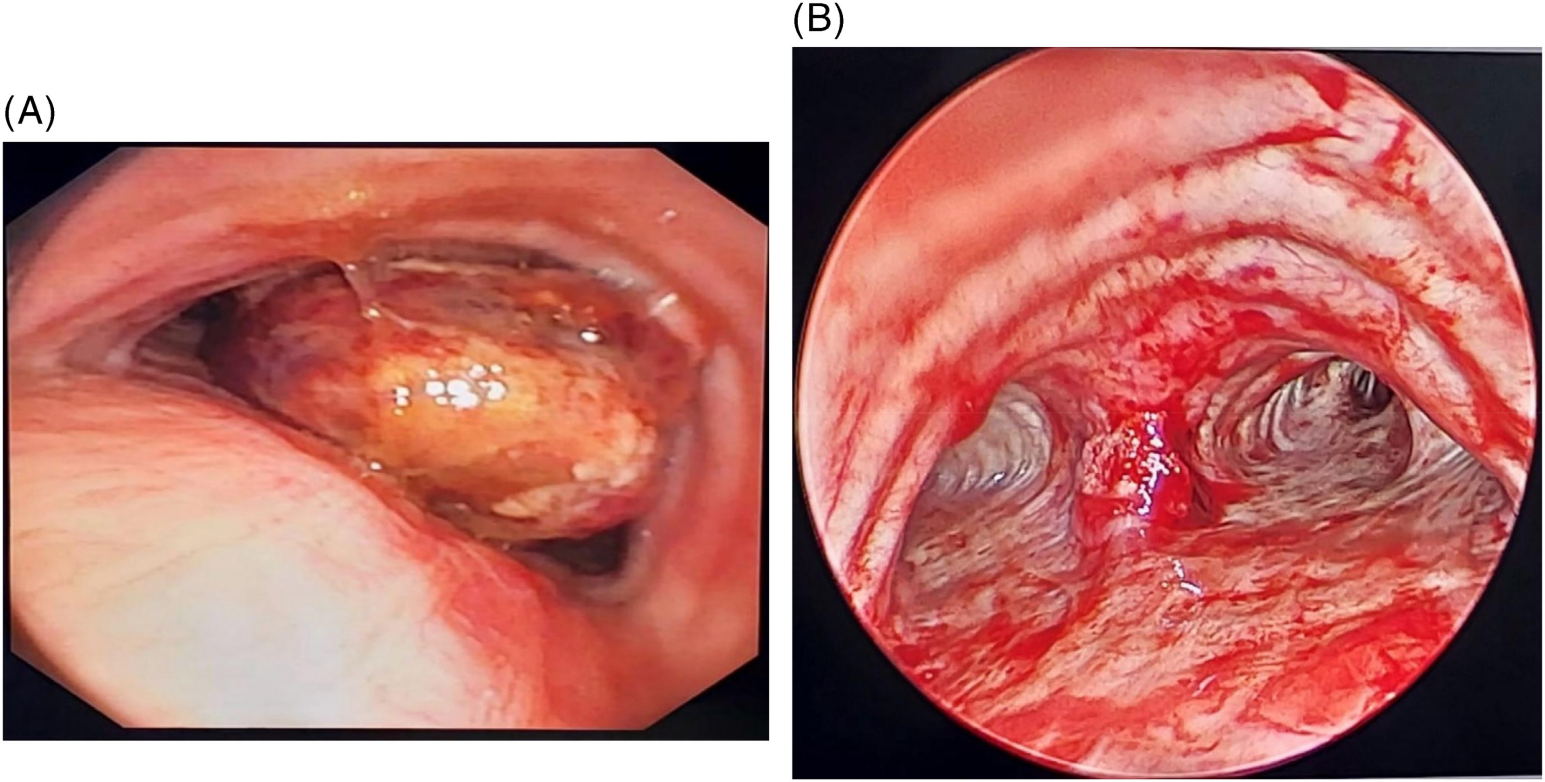
(A) Pre procedure flexible bronchoscopy demonstrated a large carinal mass arising precisely from the carina, causing more than 80% luminal occlusion of both main bronchi. (B) Post procedure follow‐up flexible bronchoscopy revealed complete recanalization with no evidence of residual disease.

Flexible bronchoscopy demonstrated a large carinal mass arising precisely from the carina, causing more than 80% luminal occlusion of both main bronchi (Figure 2). The lesion appeared narrow‐based rather than broad‐based. Given the critical airway compromise, endobronchial debulking was performed using rigid tracheoscopy. Electrocautery with a snare was initially used to snare the tumour, following which the mass was extracted using cryoextraction. Argon plasma coagulation (APC) was subsequently applied to ablate the tumour base to achieve haemostasis and reduce the risk of residual disease (Figure 2B). The retrieved tissue was submitted for histopathological and immunohistochemical evaluation.

Gross examination showed multiple grey‐white firm tissue fragments, aggregating to 3.5 × 3.1 × 1 cm.

**FIGURE 3:**
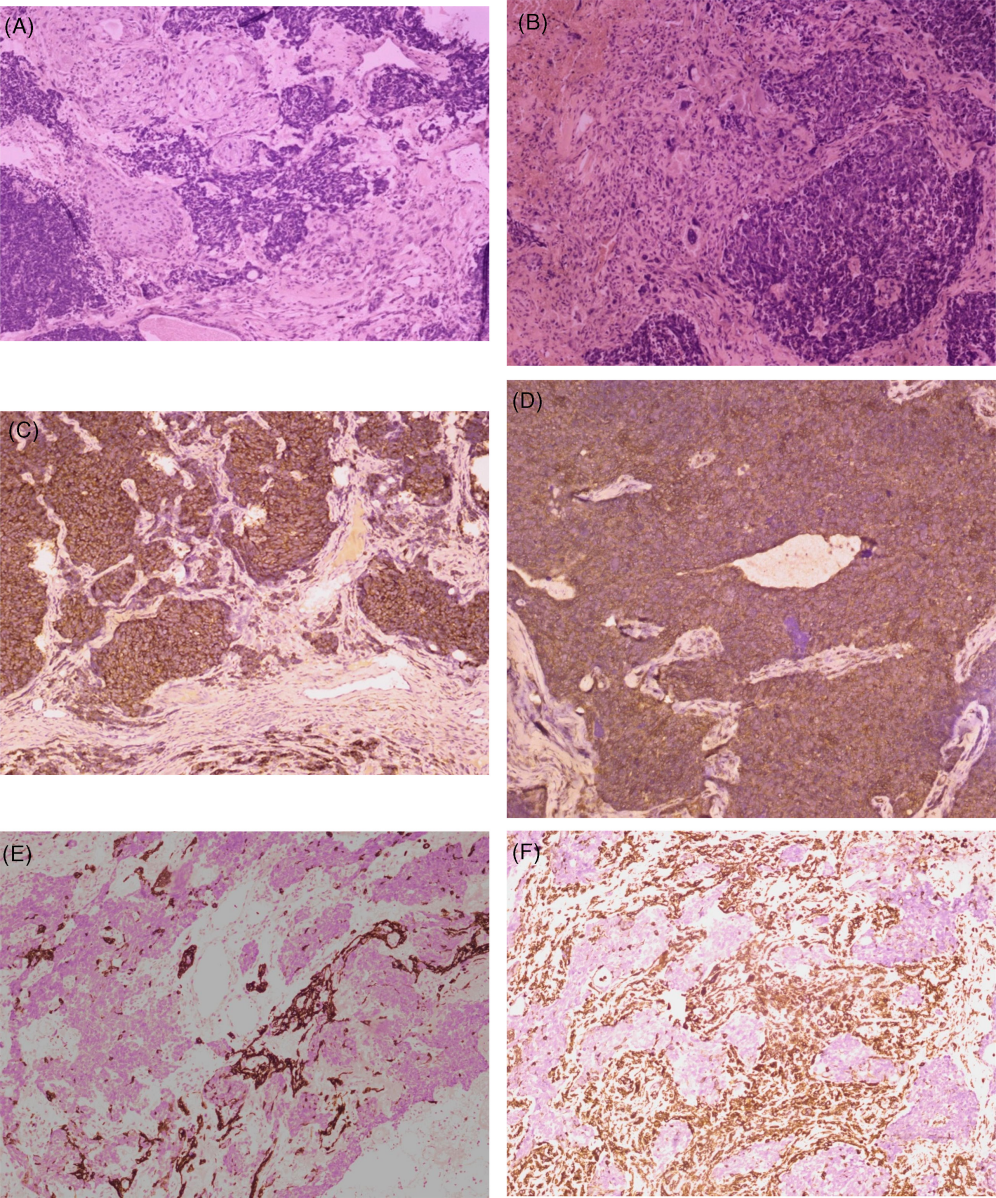
(A) Haematoxylin and Eosin (H&E) in 200X objective showed epithelial and neuroectodermal components. Epithelial islands comprised dysplastic squamous epithelium and atypical glandular structures. Intervening small blue cells with high N:C ratio, coarse chromatin, and scant cytoplasm represented the neuroectodermal component. (B) H&E (200×) showed sarcomatous component along with the neuroectodermal component. The sarcomatous areas were composed of sheets of spindle cells exhibiting marked nuclear pleomorphism, hyperchromatic nuclei, and occasional bizarre tumour giant cells. (C) Immunohistochemistry (IHC) staining (200X) for synaptophysin showed diffuse cytoplasmic granular positivity in the neuroectodermal component, while the other tumour components were negative. (D) IHC staining for CD99 (200X) showed diffuse membranous positivity in the neuroectodermal component. (E) IHC staining for GFAP (200X) showed cytoplasmic positivity in the focal neuroectodermal component, suggesting a glial differentiation. (F) IHC staining for Desmin (200X) showed diffuse cytoplasmic positivity in sarcomatous areas exhibiting skeletal muscle differentiation.

Microscopy revealed a markedly heterogeneous tumour composed of epithelial, neuroectodermal, and mesenchymal components. The epithelial (carcinomatous) component consisted of lobules, nests, and islands of dysplastic squamous cells with focal keratin pearl formation. Foci of atypical glandular structures lined by cuboidal to columnar epithelium were also identified. The neuroectodermal (teratoid) component was composed of sheets of large cells with focal rosette formation, exhibiting a high nuclear‐to‐cytoplasmic ratio, coarse chromatin, and scant cytoplasm (Figure 3A). The mesenchymal (sarcomatous) component consisted of fibro‐collagenous stroma with areas showing bizarre, pleomorphic tumour cells. Brisk mitotic activity, including atypical mitoses, along with focal necrosis and hyalinization were noted (Figure 3B).

On immunohistochemistry, epithelial membrane antigen (EMA) and pan‐cytokeratin (pan CK) showed positivity in both squamous and glandular epithelial components. The neuroectodermal component was positive for synaptophysin (Figure 3C), chromogranin, Cluster of Differentiation 99 (CD99) (Figure 3D), Cluster of Differentiation 56 (CD56), and neuron‐specific enolase (NSE), with focal Glial Fibrillary Acidic Protein (GFAP) expression showing glial differentiation (Figure 3E). The sarcomatous component showed immunoreactivity for vimentin, desmin (Figure 3F), and myogenin, confirming skeletal muscle with rhabdomyoblastic differentiation.

Octamer‐binding transcription factor ¾ (OCT3/4) and Thyroid Transcription Factor‐1 (TTF‐1) were negative, excluding a germ cell neoplasm and primary pulmonary carcinomas. Based on the characteristic triphasic morphology and supportive immunoprofile, a diagnosis of TCS was rendered.

PET‐CT demonstrated a metabolically active residual subcarinal lesion with delayed SUV increase, along with bilateral hilar, paratracheal, and pretracheal lymphadenopathy, consistent with nodal metastasis.

The patient underwent radiotherapy (50 Gy in 30 fractions) with concurrent chemotherapy using paclitaxel (50 mg) and carboplatin (AUC 2) for five cycles. At six‐month follow‐up, repeat bronchoscopy showed no local recurrence, and PET‐CT demonstrated no progression of nodal disease. The patient remains asymptomatic and under surveillance for 4 years, with no evidence of disease progression.

## 3 Discussion

TCS is an exceptionally rare and highly aggressive malignancy composed of epithelial, mesenchymal, and neuroectodermal elements. These tumours most commonly arise in the nasal cavity and paranasal sinuses, and their aggressive nature necessitates early diagnosis and multimodality treatment to improve outcomes. The histogenesis of TCS remains uncertain, with proposed origins including germ cells, pluripotent epithelial stem cells, or divergent differentiation within high‐grade neuroectodermal tumours [3, 14, 15, 16, 17, 18].

The entity was first described by Shanmugaratnam as “teratoid carcinosarcoma” [17]. Heffner et al. reviewed 20 cases and introduced the term TCS, emphasizing its complex cytoarchitecture [3].

The largest systematic review to date analysed 127 cases of sinonasal TCS, demonstrating a strong male predominance (83%) and a mean age of 50 years. With an average follow‐up of 21 months, the recurrence rate was 38%, and the two‐year survival rate was approximately 55% [19]. Notably, all survival data derive exclusively from sinonasal cases, as no large series exist for extra‐sinonasal TCS.

The non‐sinonasal TCS is exceptionally rare; the literature is largely limited to single case reports, and true case series outside the sinonasal tract comprise only very small numbers. The largest reported series includes just two cases arising in the posterior pharyngeal wall [20]. Isolated cases have been reported in the female reproductive tract, hypopharynx, cheek, oral cavity, thyroid, and intracranial region [5, 6, 7, 8, 9, 10, 11, 12, 13]. Rare congenital presentations as a nasopharyngeal mass [21] and paediatric cases have also been described [22, 23, 24]. However, to date, no primary tracheal, carinal, or bronchial TCS has been reported, rendering the present case unique.

Carinal tumours pose significant therapeutic challenges due to their critical anatomical location. Squamous cell carcinoma and adenoid cystic carcinoma are the most common malignancies encountered in this region [25]. Diagnosis of TCS in the carina relies on identifying the characteristic triphasic cellular components, supported by immunohistochemistry, and excluding other neoplasms such as squamous cell carcinoma, adenoid cystic carcinoma, small cell carcinoma, carcinoid tumours, germ cell tumours, carcinosarcoma, and primary sarcomas.

The marked histologic heterogeneity of TCS contributes significantly to diagnostic difficulty, particularly on small biopsies. In Heffner's series, many cases were initially misdiagnosed as adenocarcinoma, olfactory neuroblastoma, fibrosarcoma or rhabdomyosarcoma [3]. Most reported cases demonstrate squamous carcinoma with or without glandular differentiation, admixed with primitive neuroepithelial tissue showing rosettes and neurofibrillary matrix, and a sarcomatous component often with heterologous differentiation [26, 27, 28, 29]. Our case exhibited all three components, including rhabdomyosarcomatous differentiation.

The relevant immunohistochemical markers in the various components of TCS are listed in Table 1 [1, 28, 29, 30, 31, 32, 33, 34].

**TABLE 1:** The relevant IHC markers in various components [1, 28, 29, 30, 31, 32, 33, 34].

Tumour component	IHC markers
Epithelial	AE1/AE3, EMA, CK5/6, CK7, p63
Mesenchymal	Vimentin, Desmin, SMA, Myogenin, MyoD1, S100
Neuroectodermal	Synaptophysin, CD56, Chromogranin, NSE, CD99, GFAP.
Germ cell markers	OCT3/4, PLAP, AFP (all negative)
SWI/SNF	SMARCA4 (loss), INI1 (retained)
Wnt pathway	β‐catenin (nuclear)
Proliferation	Ki‐67 (high)

Recent molecular studies have identified recurrent alterations involving the SWI/SNF chromatin‐remodelling complex, particularly SMARCA4 inactivation, and activation of the Wnt/β‐catenin pathway. Less frequent alterations include ARID1A mutations, rare DICER1 hotspot mutations, and occasional PIK3CA mutations [30, 31, 32, 33, 34].

Given its aggressive behaviour, treatment is multimodal, incorporating surgery where feasible, radiotherapy, and platinum‐based chemotherapy. Radical surgery followed by radiotherapy remains the most common approach [35, 36]. In the largest treatment review, surgery with radiation therapy was employed in 59.3% of cases [37]. Chapurin et al. reported that most patients received combined modalities, including surgery with adjuvant chemoradiation [19]. Non‐surgical chemoradiotherapy has also been reported with short‐term disease control in select cases [38].

In the present case, life‐threatening airway obstruction necessitated urgent bronchoscopic debulking, followed by definitive chemoradiotherapy, resulting in sustained disease control.

This appears to be the first documented case of TCS presenting as a carinal mass with bilateral bronchial occlusion. Prompt bronchoscopic debulking followed by combined chemoradiotherapy resulted in favourable clinical and endoscopic outcomes. This case expands the anatomical spectrum of teratocarcinosarcoma, highlighting the potential for occurrence at unusual sites and underscoring the risk of underdiagnosis on limited biopsies due to marked histologic heterogeneity. Adequate tissue sampling, comprehensive immunohistochemical evaluation, and a multimodality treatment approach are essential for accurate diagnosis and effective disease control.

## Author Contributions

Gittwa Kottangal contributed to the histopathological examination and immunohistochemical analysis, and provided a definitive pathological diagnosis and did a literature review. Although based at Aster MIMS Calicut, the pathology departments of Aster MIMS Calicut and Aster MIMS Kannur function collaboratively; therefore, she was directly involved in the diagnostic workup of this case. Vishnu Gopalakrishnan was involved in the clinical evaluation, performed the bronchoscopic intervention and endobronchial debulking, supervised patient management, and critically reviewed the manuscript. Binil Salam participated in the bronchoscopic procedure, contributed to peri‐procedural decision‐making, and reviewed the manuscript. Avinash Murugan assisted in the interventional procedure, contributed to patient management planning, and reviewed the manuscript for intellectual content. Supriya Ranjith provided anaesthetic management and peri‐procedural support during the intervention. Pramathakala Mettak assisted in the bronchoscopic procedure and contributed to clinical data collection. Vishnu Suresh (Internal Medicine Resident, posted in Pulmonology, Aster MIMS Kannur) assisted in patient care, compiled clinical and imaging data, contributed to the literature review, and assisted in drafting the initial manuscript.

## Funding

The authors have nothing to report.

## Consent

The authors declare that written informed consent was obtained for the publication of this manuscript and accompanying images using the consent form provided by the Journal.

## Conflicts of Interest

The authors declare no conflicts of interest.

## Data Availability

The data that support the findings of this study are available on request from the corresponding author. The data are not publicly available due to privacy or ethical restrictions.
